# Supplementary material for: An all-atom protein generative model
Source: bioRxiv. 2023 May 25:2023.05.24.542194. Preprint. [Version 1] doi: 10.1101/2023.05.24.542194 (PMC10245864; doi:10.1101/2023.05.24.542194)
Supplement: Supplement 1 [file NIHPP2023.05.24.542194v1-supplement-1.pdf]

A.4 Supplemental Figures

bioRxiv preprint doi: https://doi.org/10.1101/2023.05.24.542194; this version posted May 25, 2023. The copyright holder for this preprint (which was not certified by peer review) is the author/funder, who has granted bioRxiv a license to display the preprint in perpetuity. It is made available under aCC-BY 4.0 International license.

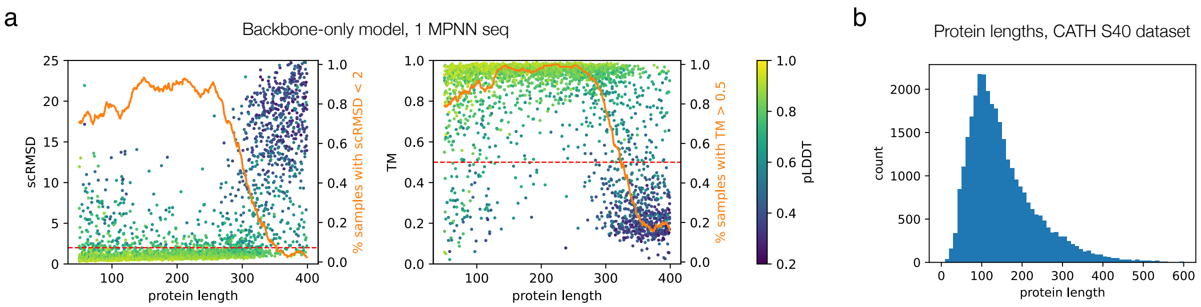

Figure S1. Additional metrics. **(a)** Self-consistency metrics for backbone samples as in Fig. 1, but with only one ProteinMPNN-designed sequence per backbone. **(b)** Distribution of protein lengths in the dataset (excluding proteins with length > 600).

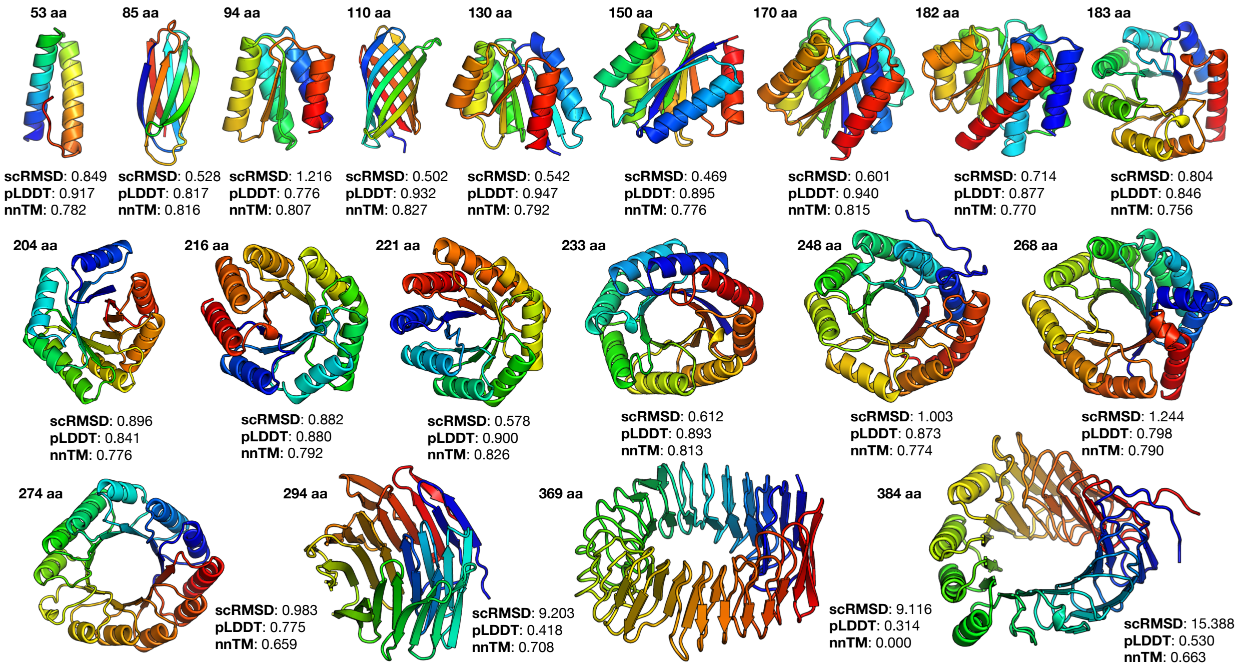

Figure S2. Samples from backbone Protpardelle. Non-cherry-picked raw samples from the backbone model. scRMSD is the best out of 8 ProteinMPNN sequences with ESMFold, with the corresponding pLDDT. Where nnTM is zero, it means we did not find any matches with FoldSeek.

bioRxiv preprint doi: https://doi.org/10.1101/2023.05.24.542194; this version posted May 25, 2023. The copyright holder for this preprint (which was not certified by peer review) is the author/funder, who has granted bioRxiv a license to display the preprint in perpetuity. It is made available under aCC-BY 4.0 International license.

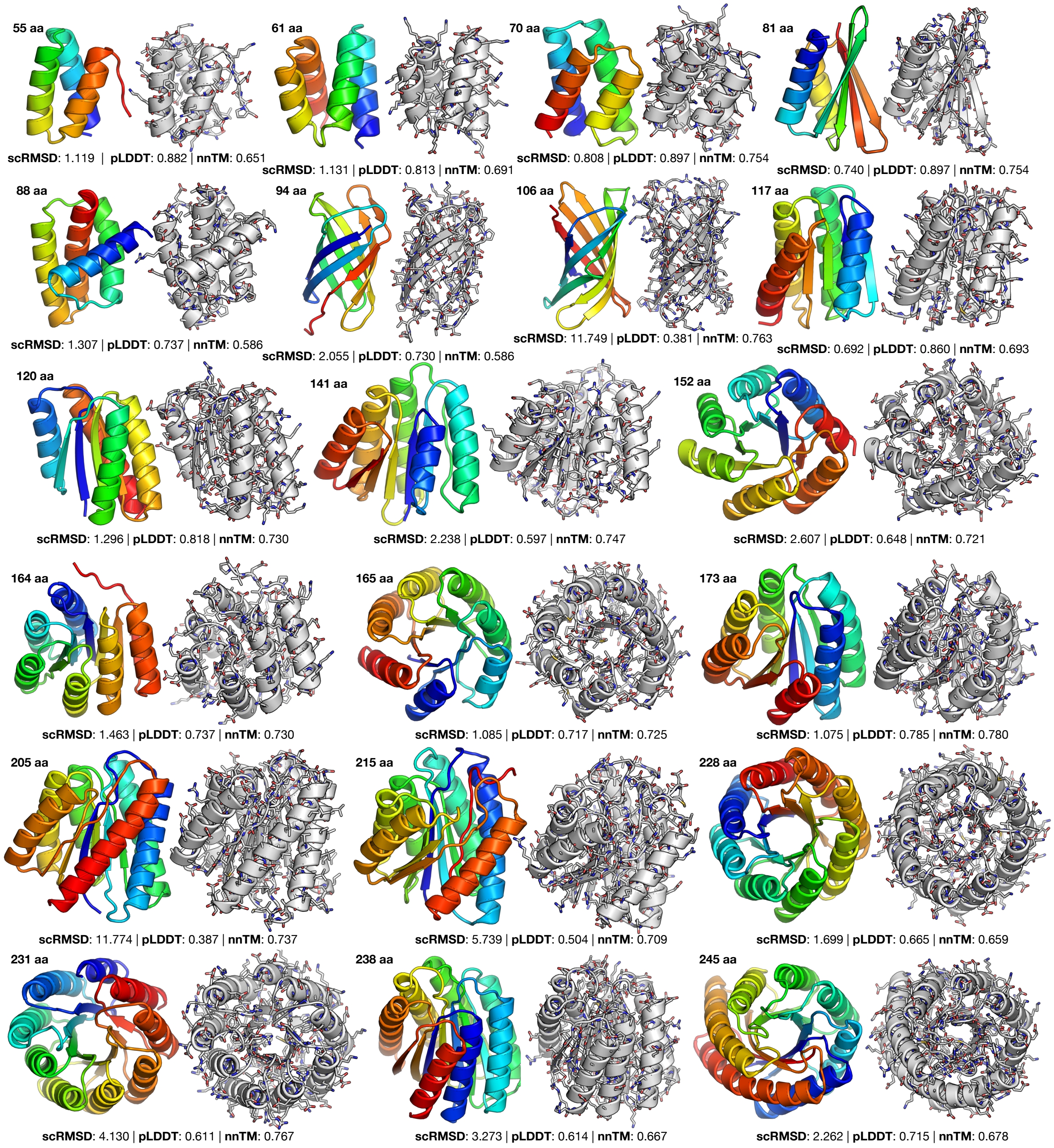

Figure S3. Samples from all-atom Protpardelle. Non-cherry-picked raw samples from the all-atom model. scRMSD is the best out of 8 ProteinMPNN sequences with ESMFold, with the corresponding pLDDT. The structure without sidechains is shown in color, and the same structure with sidechains shown adjacent without color.

bioRxiv preprint doi: https://doi.org/10.1101/2023.05.24.542194; this version posted May 25, 2023. The copyright holder for this preprint (which was not certified by peer review) is the author/funder, who has granted bioRxiv a license to display the preprint in perpetuity. It is made available under aCC-BY 4.0 International license.

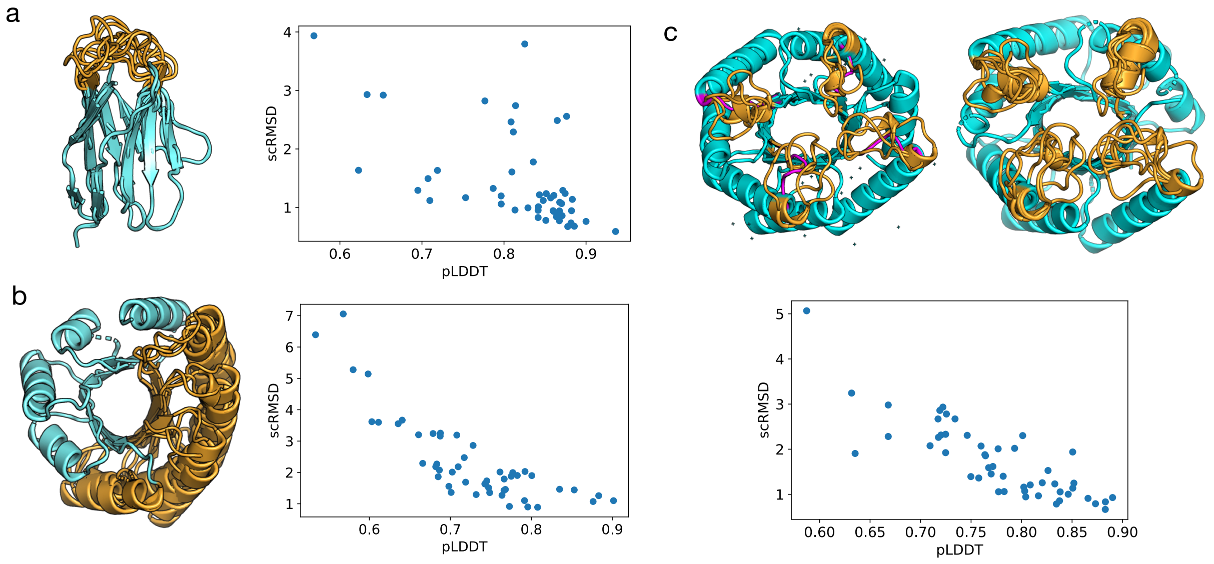

Figure S4. Inpainting ensembles. Inpainting ensembles for **(a)** a loop of a monobody (PDB: 5X2O, chain L) **(b)** half of a *de novo* TIM barrel (PDB: 5BVL) and **(c)** four loops of a *de novo* ovoid TIM barrel (PDB: 7UEK). The conditioning portion is shown in blue, the model generated portion in brown, and the magenta is the original loop. The inpainted structure in (a) and (b) demonstrate same-length inpainted structure, while the length of the loops in (c) are each four residues longer than the original loop length. Sequences were designed with ProteinMPNN and predicted with ESMFold; design success is shown in the plots.

bioRxiv preprint doi: https://doi.org/10.1101/2023.05.24.542194; this version posted May 25, 2023. The copyright holder for this preprint (which was not certified by peer review) is the author/funder, who has granted bioRxiv a license to display the preprint in perpetuity. It is made available under aCC-BY 4.0 International license.

**model**	**sampling**	**runtime** (s)	**steps**	**schurn**	**step scale**	**scRMSD** ($\mu \pm \sigma$)	**count**	**bond metric**
amber-grass-27, ep1900	2 for 50:128:1	1371.13	1000	200	1	9.70 ± 10.82	156	0.11
amber-grass-27, ep1900	2 for 50:128:1	1336.85	1000	200	1.2	6.19 ± 8.43	156	0.09
amber-grass-27, ep1900	2 for 50:128:1	1341.57	1000	200	1.5	5.66 ± 7.57	156	0.08
amber-grass-27, ep1900	2 for 50:128:1	1617.17	1000	400	1	8.00 ± 9.91	156	0.11
amber-grass-27, ep1900	2 for 50:128:1	1359.52	1000	400	1.2	4.64 ± 6.62	156	0.08
amber-grass-27, ep1900	2 for 50:128:1	1353.33	1000	400	1.5	4.27 ± 5.74	156	0.07
amber-grass-27, ep1900	2 for 50:128:1	1857.78	1500	400	1.2	4.83 ± 7.13	156	0.08
amber-grass-27, ep1900	2 for 50:128:1	2418.27	2000	400	1.2	8.81 ± 11.33	156	0.20
amber-grass-27, ep1900	2 for 50:128:1	1003.19	500	200	1.2	3.20 ± 4.57	156	0.08
amber-grass-27, ep1900	2 for 50:256:1	3683.36	1000	100	1	23.57 ± 17.56	412	0.16
amber-grass-27, ep1900	2 for 50:256:1	3609.14	1000	100	1.2	20.98 ± 18.66	412	0.12
amber-grass-27, ep1900	2 for 50:256:1	3538.07	1000	100	1.5	15.98 ± 18.13	412	0.09
amber-grass-27, ep1900	2 for 50:256:1	1209.80	200	0	1	15.59 ± 11.51	412	0.16
amber-grass-27, ep1900	2 for 50:256:1	1058.20	200	0	1.2	11.93 ± 14.31	412	0.12
amber-grass-27, ep1900	2 for 50:256:1	1093.13	200	0	1.5	16.43 ± 12.69	412	0.15
amber-grass-27, ep1900	2 for 50:256:1	1305.67	200	100	1	14.56 ± 13.73	412	0.17
amber-grass-27, ep1900	2 for 50:256:1	1256.96	200	100	1.2	10.05 ± 13.69	412	0.17
amber-grass-27, ep1900	2 for 50:256:1	1224.22	200	100	1.5	23.32 ± 13.51	412	0.31
amber-grass-27, ep1900	2 for 50:256:1	1266.96	200	200	1	14.93 ± 13.39	412	0.16
amber-grass-27, ep1900	2 for 50:256:1	1232.18	200	200	1.2	10.11 ± 13.76	412	0.17
amber-grass-27, ep1900	2 for 50:256:1	1237.49	200	200	1.5	22.75 ± 13.31	412	0.33
amber-grass-27, ep1900	2 for 50:256:1	1326.97	200	50	1	None	None	0.17
amber-grass-27, ep1900	2 for 50:256:1	1255.39	200	50	1.2	10.58 ± 13.61	412	0.15
amber-grass-27, ep1900	2 for 50:256:1	1288.46	200	50	1.5	19.16 ± 14.53	412	0.28
amber-grass-27, ep1900	2 for 50:256:1	2046.42	500	0	1	16.73 ± 13.99	412	0.17
amber-grass-27, ep1900	2 for 50:256:1	2004.81	500	0	1.2	12.45 ± 15.69	412	0.10
amber-grass-27, ep1900	2 for 50:256:1	1971.94	500	0	1.5	15.42 ± 15.09	412	0.10
amber-grass-27, ep1900	2 for 50:256:1	2266.17	500	100	1	18.28 ± 16.92	412	0.15
amber-grass-27, ep1900	2 for 50:256:1	2117.65	500	100	1.2	14.93 ± 18.06	412	0.19
amber-grass-27, ep1900	2 for 50:256:1	2171.80	500	100	1.5	13.93 ± 18.20	412	0.09
amber-grass-27, ep1900	2 for 50:256:1	2136.91	500	200	1	17.86 ± 16.92	412	0.15
amber-grass-27, ep1900	2 for 50:256:1	2147.56	500	200	1.2	12.47 ± 17.54	412	0.19
amber-grass-27, ep1900	2 for 50:256:1	2115.05	500	200	1.5	11.42 ± 16.11	412	0.09
icy-glade-31, ep850	4 for 50:256:1	2822.46	500	200	1.2	5.46 ± 5.45	824	0.12
lemon-shape-51, ep1200	8 for 50:256:1	3683.67	500	200	1.2	4.47 ± 3.78	1300	0.11
**lemon-valley-40**, ep2650	8 for 50:256:1	3523.56	500	200	1.2	3.46 ± 4.33	1648	0.11
rare-valley-39, ep1400	16 for 50:256:1	7774.16	500	200	1.2	4.85 ± 4.16	3296	0.11
rich-tree-35, ep1100	4 for 50:256:1	3161.89	500	200	1.2	5.00 ± 4.22	824	0.11
rich-tree-35, ep1300	4 for 50:256:1	2764.66	500	200	1.2	4.26 ± 3.56	824	0.11

Table 1: Experiments on sampling hyperparameters for the all-atom model. For all all-atom results in this paper, we use the "lemon-valley-40, ep2650" model described here. "sampling" describes how many proteins were sampled for each length; the lengths are described in slice format, i.e. 2 for 50:128:1 indicates that 2 proteins at each length from 50 to 128 were sampled. "runtime" describes the number of seconds used to sample all proteins in this set (the number of proteins generated is given in "count"). "steps" describes the number of ODE discretization steps. "schurn" describes the amount of noise that was injected during sampling. "step scale" describes the scale applied to the score before multiplying by the step size. "scRMSD" is the mean and std of scRMSD for all samples in this experiment. "count" describes the total number of proteins sampled. "bond metric" is the bond length RMSE over all atoms.
